# Spontaneous speech feature analysis for alzheimer's disease screening using a random forest classifier

**DOI:** 10.3389/fdgth.2022.901419

**Published:** 2022-11-17

**Authors:** Lior Hason, Sri Krishnan

**Affiliations:** Department of Electrical, Computer, and Biomedical Engineering, Toronto Metropolitan University, Toronto, Ontario, Canada

**Keywords:** Alzheimer's disease, spontaneous speech, machine learning, classification, acoustic features, non-stationarity, non-linearity, data augmentation

## Abstract

Detecting Alzheimer's disease (AD) and disease progression based on the patient's speech not the patient's speech data can aid non-invasive, cost-effective, real-time early diagnostic and repetitive monitoring in minimum time and effort using machine learning (ML) classification approaches. This paper aims to predict early AD diagnosis and evaluate stages of AD through exploratory analysis of acoustic features, non-stationarity, and non-linearity testing, and applying data augmentation techniques on spontaneous speech signals collected from AD and cognitively normal (CN) subjects. Evaluation of the proposed AD prediction and AD stages classification models using Random Forest classifier yielded accuracy rates of 82.2% and 71.5%. This will enrich the Alzheimer's research community with further understanding of methods to improve models for AD classification and addressing non-stationarity and non-linearity properties on audio features to determine the best-suited acoustic features for AD monitoring.

## Introduction

Alzheimer's disease (AD) is a neurodegenerative disease that affects cognitive functions such as speech and language. AD is a progressive kind of dementia that gets worse over time. Dementia is a broad term that refers to conditions that affect memory, thinking, and behavior because of brain damage or diseases (1–3), and is a serious public health problem that affects 5% to 10% of the older population (4). There are over 46.8 million people living with Alzheimer's disease or other forms of dementia worldwide, with over 10 million new cases diagnosed each year, meaning one new case every 3.2 s. By 2030, the number will have risen to 78 million, and by 2050, it will have risen to 139 million. China, India, and their south Asian and western Pacific neighbors have the fastest-growing elderly populations (5). Only 45 percent of Alzheimer's patients are informed of their diagnosis. As a result, early detection is critical (5, 6). Early and accurate diagnosis, according to a 2018 study, might save up to $7.9 trillion in medical and care expenses (7).

Due to a lack of a clear diagnosis and feasible curative treatments, it is difficult to fight this disease because once it reaches the latest stages, it is difficult to prepare properly and therefore this disease has become a serious public health issue, prompting research into non-drug-based solutions. Among these techniques, speech processing has proven to be a vital and an emerging research topic (8). Difficulties in producing and understanding speech are linked to memory functions in Alzheimer's patients (9). Machine learning (ML) algorithms use biomedical research to classify diseases and provide a better knowledge of it. ML classifiers have been shown to be useful in the diagnosis of AD using several types of data, including clinical and neuropathological research data, MRI brain imaging, and even pathological speech of AD patients (8, 10). Data augmentation techniques were used to increase the amount of training data, avoiding overfitting, and improving the model's robustness. There is a need for cost-effective and repetitive monitoring methods for early detection of AD and prediction of disease progression. Classification based on acoustic features only was attempted in (11) and in (12) with the IS10-Paralinguistics feature set which includes Mel-frequency cepstral coefficients (MFCC), speech duration, pause-related features, pitch-related features, and other prosodic features. They employed a logistic regression classifier with leave-one-subject-out (LOSO) cross-validation and a support vector machine classifier (SVC) with a linear kernel and obtained an accuracy rate of 76.85%. Another work (13) used the Dementia Bank dataset, relied exclusively on acoustic features, and a classification accuracy achieved was 94.71% using the Bayes Net (BN) classifier, where 263 features of the audio files were extracted and the top 20 features among them were selected.

The ADReSS dataset presents a more challenging and enhanced spontaneous speech dataset, as well as requires the building of models directly from speech without the use of manual transcription (14). In a recent study (11), acoustic features from the eGeMAPS (15–17) feature set containing frequency-related parameters, energy/amplitude-related parameters, and spectral (balance) parameters, totaling 88 features per 100 ms frame were used. The silence ratio was discovered to be more important than other linguistic variables. For each audio recording, they used the active data representation method (ADR) (18) to produce a frame-level acoustic representation. In LOSO cross-validation (CV), the best classifier was the decision tree (DT), which achieved 78.87% and 72.89% accuracy rates using acoustic and linguistic characteristics, respectively. Using linguistic features involves the use of either low-accuracy automatic speech recognition (ASR) technologies or transcription methods, both of which are inconvenient for patients, can be costly, and time-consuming (19). Some linguistic characteristics are influenced by both content and language. Although there are some linguistic aspects that are not content-dependent, such as word frequency measurements, extracting content-independent linguistic features is challenging to automate (20). The use of acoustic and context-free language features to categorize patients has yielded encouraging findings for AD detection (21).

The focus of this paper is to analyze the stationarity and linearity properties of spontaneous speech, extracting acoustic features with similar properties for predicting early AD diagnosis and prognosis. This project focuses on improving the results of the AD classification task and identifying the stages of AD with various methods using ML approaches.

## Materials and methods

### Dataset

The ADReSS Challenge dataset consisting of 157 audio files from male and female participants was used. These audio files are recordings of picture descriptions, which have been produced by cognitively normal subjects (“CN’) and subjects suffering from Alzheimer's (“AD”), all of whom were asked to participate in a standard cognitive test for dementia known as the Cookie Theft picture test from the Boston Diagnostic Aphasia Exam (14). On an average, the length of each signal is approximately 80 s. Participants were shown a picture of a woman washing dishes in an overflowing sink and two children taking cookies from a jar in this test. Patients were asked to describe what they observe, and medical specialists examine their speech patterns to determine whether they have AD. Access to the ADReSS dataset was requested from the Alzheimer's Dementia Recognition through Spontaneous Speech: The ADReSS Challenge website (22).

### Model

**Figure 1:**
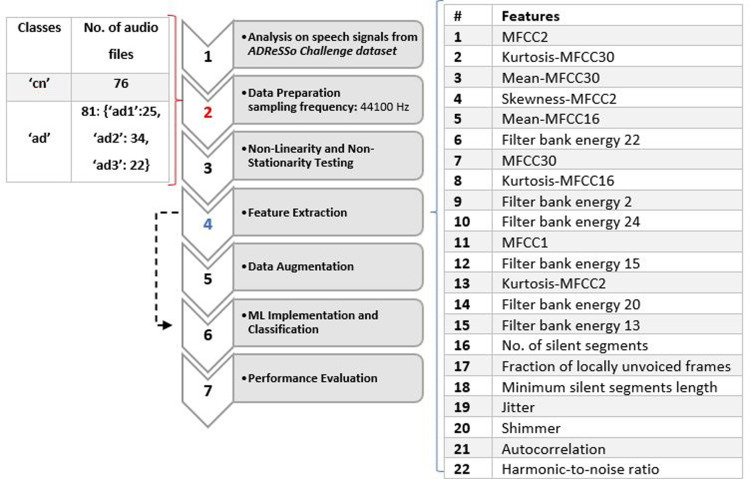
Flowchart diagram of the proposed system with list of features used and dataset details.

The proposed system was divided into seven main parts: Non-Linearity and Non-Stationarity Testing, Data Representation and Feature Analysis, Cross-Validation, Data Augmentation and ML Classification. Classifiers were evaluated using data set partitioning during a 10-fold cross-validation process. The first task involved AD classification with 2 classes (cn and ad), and the second task involved AD stages classification with 4 classes (cn, ad1, ad2, ad3) that were separated based on observation only. The number of samples per class are as follows: {“cn”:76, “ad”:81 [“ad1′:25, “ad2′:34, “ad3′: 22]}. The number of samples for the AD stages classification was divided based on auditory observations. For the early stage (ad1) class, audio files were chosen from the 81 “ad” class based on the level of participants' rich vocabulary, and clarity, and participated in a meaningful conversation with the interviewer. They had an easier time expanding on the description of the cookie theft picture. In some cases, participants were challenged to find the right words and answered slower than normal (cn) participants. Participants in the intermediate (ad2) and advanced (ad3) stages were characterized based on the following criteria: Had more difficulty finding the right word, used familiar words repeatedly, described familiar objects rather than calling them by name, lost their train of thoughts easily, and had difficulty organizing words logically. Subjects who had longer pauses, slurred and unclear speech with a higher frustration were classified as advanced (23). See Figure 1 for more detail.

### Non-Linearity and non-stationarity testing

Typically, an audio signal is hypothesized as non-stationary and non-linear. Analyzing the speech signals from the ADReSS dataset confirmed this. If the first order (e.g., mean value) and second-order statistics (e.g., variance) of a signal do not change over time, it is said to be stationary (24, 25). Like most biomedical signals, voice signal frequency contents will include a variety of components, and these components will change over time (24). Various tests were performed to confirm that speed signals are non-linear and non-stationary. The first test involved analyzing the transition rate of root mean square (RMS) values combined with the transition rate of spectral parameters such as Line Spectrum Frequencies (LSFs) in non-stationarity testing. The second test is the Brock-Dechert-Scheinkman (BDS) test (26) which is a nonparametric test of the null hypothesis that the data is distributed independently and identically against an unnamed alternative. *p*-values of less than 0.05 suggest that the data does not follow the normal distribution. It indicates convincing evidence to reject the null hypothesis, and *p*-value below 0.05 indicates significant non-linearity (27). It was proven that most of the audio signals from the dataset have *p* values that are less than 0.05. The *p*-value for all the audio signals is 0.005. The third test involved measuring the degree of non-linearity using the nonlinear autoregressive with external model input (ARX) with a model order of 25. Larger detection ratio values (>2) indicate that a significant nonlinearity was detected, smaller values (0.5) indicate that no significant nonlinearity was detected and that any error not explained by the linear model is mostly noise, and values close to 1 indicate that the nonlinearity detection test is unreliable and that a weak nonlinearity may exist (28). An order of 25 was chosen because when plotting speech signals on spectrograms, there are 13 peaks on the positive frequency and the order of the signal model could be defined as *2n-1*, where *n* is the number of peaks. When testing the whole duration of the signal, non-linearity results were inconsistent and it was found that after segmenting the signal into 20-second-long durations, it was evident that non-linearity was detected. The average detection ratio for “CN” signals was 13.2%, for “AD1′: 8.5%, for “AD2′:9.6% and for “AD3′: 11.6%. These findings suggest that normal speech is more non-linear and dynamic than AD speech. However, further testing on a larger dataset should be conducted to conclude these findings.

### Data representation and feature analysis

**Figure 2:**
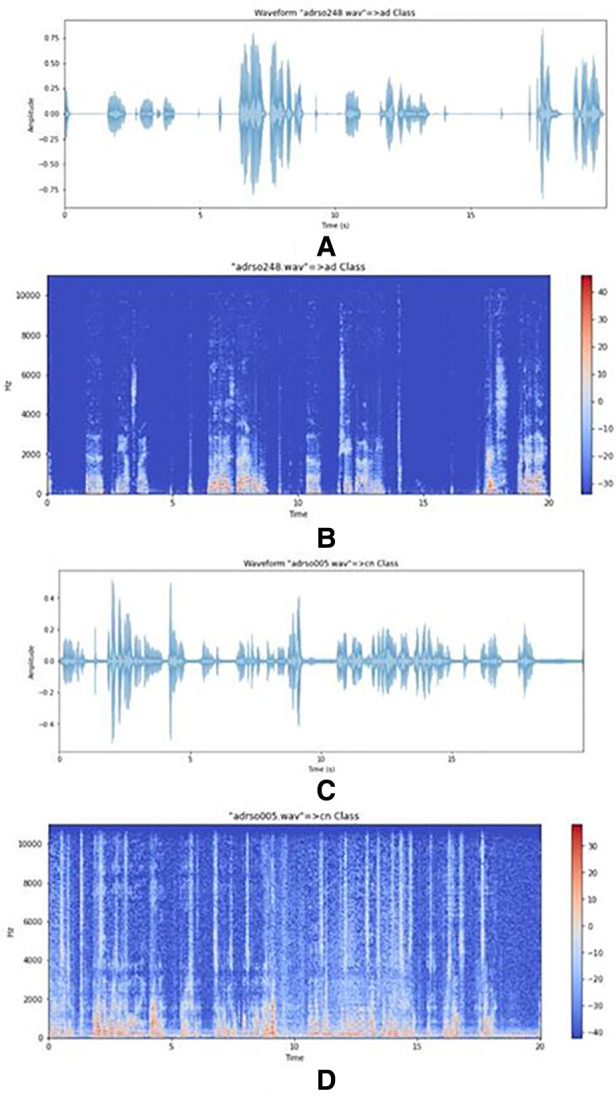
(**A**) plot of an AD speech signal. (**B**) Spectrogram of an AD speech signal. (**C**) Plot of a CN signal. (**D**) Spectrogram of a CN signal.

In the data representation and visualization stage, spectrograms, and MFCCs were implemented. Small peaks are evident in the AD speech spectrogram shown in Figure 2B compared to the CN speech spectrogram shown in Figure 2D which suggests that because the volume of speech is low there is not enough resonance to produce clear, large peaks. Therefore, small noisy peaks are evident compared to CN.

In the feature extraction stage, acoustical features were extracted from both training and testing speeches. Three classifiers that were trained on the audio features are Logistic Regression, SVM and Random Forest. Acoustic features with linear and non-linear expressions were derived directly from the training data's speech. The acoustic analysis parameters offer the advantage of objectively describing the voice. The sub laryngeal and laryngeal systems are finely controlled by phonation and voice quality. Features in (13) include MFCC, kurtosis, mean, skewness, filter bank energy, number of silent segments, a fraction of locally unvoiced frames, and minimum silent segments length. Jitter, shimmer, autocorrelation, and the harmonics-to-noise (HNR) ratio were employed in (11) and (12). These acoustic features were implemented in this study, shown in Table 1.

**Table 1:** (**A**) classification accuracy for AD/CN and AD stages/CN models after applying acoustic features and data augmentation methods. (**B**) Model evaluation metrics for Random Forest classifier before and after applying data augmentation methods for Task 1. (**C**) Random Forest classier accuracy before and after applying data augmentation methods for Task 2.

Model	Accuracy (%) (Task 1)	Accuracy (%) (Task 2)
(1A)		
SVM	78.6	65.2
Logistic Regression	66.2	56.7
Random Forest	82.2	71.5
(B)		
Random Forest Classifier	Task 1 (Before applying data augmentation methods)	Task 1 (After applying data augmentation methods)
Accuracy (%)	72.6	82.2
AUC (%)	75.7	89.3
Recall (%)	73.4	81.4
Precision (%)	74.9	81.6
(C)		
Random Forest Classifier	Task 2 (Before applying data augmentation methods)	Task 2 (After applying data augmentation methods)
Accuracy (%)	46.3	71.5

### Cross-Validation

Cross-validation is a method for evaluating a predictive model that divides the original sample into a training set and a validation/test set for training and evaluating the model. It is also a good strategy to avoid model overfitting (29). The original samples are randomly partitioned into 10 parts: 9 for training and 1 for testing. This procedure was repeated 10 times where each time reserving a different tenth for testing. 10 equal-sized subsamples, and one subsample is kept as validation data for testing the model, while the remaining 9 subsamples are used as training data in 10-fold cross-validation.

### Data augmentation and ml classification

Data augmentation is a standard approach for increasing the amount of training data, helping avoid overfitting, and improving the model's robustness. For training, speech signal systems require a large database, and data augmentation can be extremely valuable when starting out with small data sets. Exposing the classifiers to alternative representations of the training samples makes the model less biased, more invariant, and robust to such transformations when attempting to generalize the model to new datasets (30–32). To obtain high accuracy and fully preserve the information inside the signal, an audio segmentation technique was applied to lower the classifier's misclassification rate. To avoid overfitting, this is especially beneficial with small, annotated datasets and complex trainable algorithms (33). The speech segmentation method involves dividing speech signals into short-time segments. On an average, the length of each signal is approximately 80 s. The non-stationarity property was managed by using fixed segments of 20 s. Each audio file was split into multiple frames to increase the number of samples in the dataset. It was found that 20 s is a good frame length because it considers sentences which is essential for acoustic feature extraction. If a smaller frame length was considered, then it may result in a few words per frame. The number of samples has now increased to 554 samples from the original 157 samples dataset. To further improve the accuracy of the model, a speech perturbation method was applied to the recordings. For each utterance in the training set, warping factors of 0.7, 0.8, 1.2, and 1.3 were chosen to warp the frequency axis to slightly distort the speed of the original speech signal and create a new replica of it. In this work, four copies of the original speech signal were added to the training set. The final number of samples after this step increased to 2811, which is approximately 18 times the original. With 10-fold cross-validation, the model was trained 10 times, each time 2811/10 samples were considered as the test set and the model is being trained based on the other remaining samples.

For both AD/CN and Stages AD/CN classifications, three classification models were considered: Logistic Regression, SVM, and Random Forest. The audio files were divided into two categories: 70% for training, and 30% for testing and validation. For the first and second tasks, the best performing classifier in cross-validation was Random Forest.

## Results

The results for AD vs. CN and AD Stages vs. CN classification tasks are summarized in Table 1. Three classifiers (Random Forest, Logistic Regression, and Support Vector Machine (SVM)) were evaluated in this study. For the first task, the best performing classifier in cross-validation was Random Forest, followed by SVM, and finally, Logistic Regression, achieving 82.2%, 78.6%, and 66.2% accuracy respectively for models containing the combination of acoustic features and data augmentation methods, respectively. For the second task, the best performing classifier in cross-validation was Random Forest, followed by SVM, and finally, Logistic Regression, achieving 71.5%, 65.2% and 56.2% accuracy respectively for a model containing the combination of acoustic features and data augmentation methods, respectively. For Tasks 1 and 2, Random Forest classifier model evaluation metrics (Accuracy (%), AUC (%), Recall (%), Precision (%)) before and after applying data augmentation methods are shown in Table 1.

**Figure 3:**
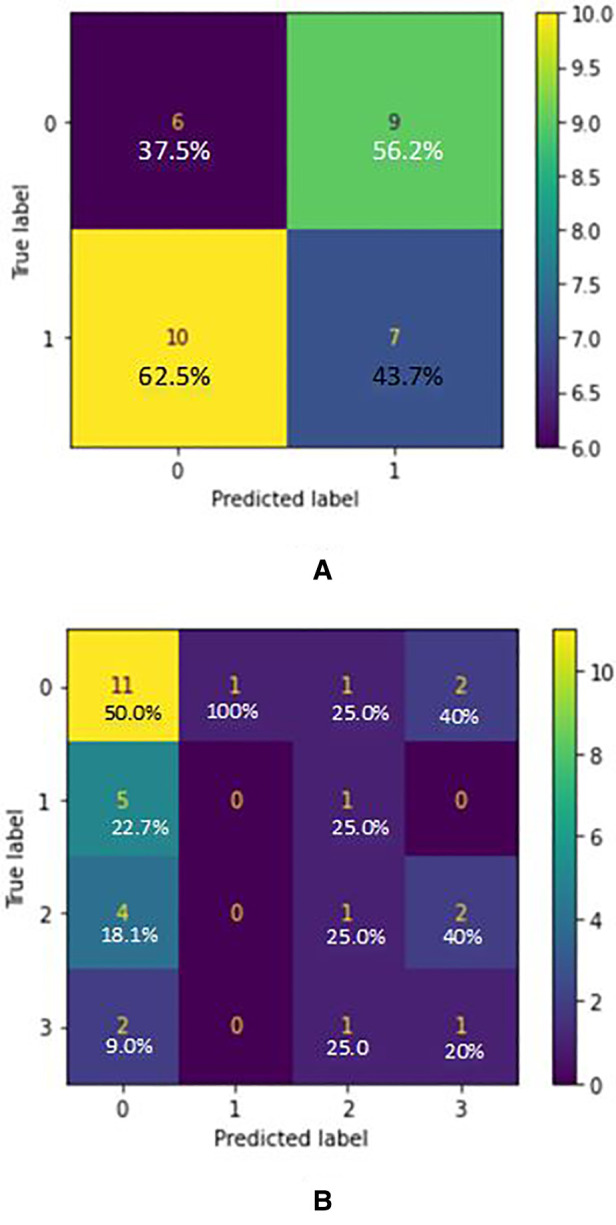
(**A**) confusion matrix for AD/CN classification (true label VS predicted label) - random forest classifier (acoustic features) where label 0 indicated “cn” and label 1 indicated “ad”. (**B**) Confusion matrix for AD Stages/CN classification (True label VS Predicted label) - Random Forest Classifier (acoustic features) where label 0 indicated “cn”, label 1 indicated “ad1”, label 2 indicated “ad2” and label 3 indicated “ad3”.

Based on the confusion matrix of the RandomForest model for AD classification in Figure 3, the predictions of the first class (CN) have 15% errors and for the AD class, it is 20% which shows that the model predicts CN samples better than the AD ones. Also, for the AD stages vs. CN, the error rate for each class is 23%, 5%, 30%, and 22.5% respectively (considering CN, AD stage 1, AD stage 2, AD stage 3). Results show that the model is best performing for the stage 1 class and has the worst performance for the stage 2 class. Figure 2 graph shows the importance of features based on the Random Forest model. Jitter and amplitude in decibels (shimmer) are higher in AD speech, and there are more speech pauses.

## Discussion

This study involves exploratory data analysis to understand why machine learning models make the decisions they do, and why it matters. It was found that typically speech signals have a non-linear and a non-stationarity property. Signal segmentation was applied as a pre-processing step for non-stationarity signal analysis. Non-linearity property was handled by applying a large portion of acoustic features with a non-linear expression. As well, unlike traditional machine learning models, deep learning models require a lot of training data. We found that the most noticeable linguistic changes in recordings of AD patients from the ADReSS Challenge dataset are longer hesitations and a decreased speech rate in spontaneous speech by listening intently to the recordings of AD patients. Articulation, fluency (word-finding ability, hypo fluency, hyper fluency), semantic fluency, and repetition were noted as linguistic measurements. Most AD patients had problems detecting and naming things when asked to describe the “Cookie Theft” image, and they used the same terminology to describe the image. According to (34, 35), these symptoms appear early in the illness and worsen with time, implying that these patients' semantic memory is impaired early on and worsens over time. Until late in the disease, the articulatory and syntactic areas of language production remain intact (36). We noticed that all Alzheimer's patients spoke slowly, with frequent pauses, and took a long time to find the right word, resulting in speech disfluency. A subset of Alzheimer's patients spoke even more slowly, with greater pauses, and some had slurred and garbled speech that was difficult to understand. Prior to data augmentation, silent segment percentage was found to be a dominant feature. It was an important feature that was used to separate the “ad” data into stages. For detecting the silence part, a detection algorithm based on thresholding method was used. To use this method, the frequency effect was omitted, and the envelope of the signal was extracted. Then the samples of the signal have been ordered in an increasing format and the average of the first 90% of the samples (of the ordered signal) has been considered as the threshold value, so the samples which were below the threshold are considered as noise samples or we can say they show the silence parts. To omit the interviewer's speech, a 10-second guard was considered and omitted from the beginning and end of the signal.

According to (37), in moderate or severe AD, there are more serious temporal abnormalities in spontaneous speech: the quantity and duration of hesitations rise when compared to mild AD, and accessing the mental lexicon becomes even more difficult. AD patients produce shorter descriptions than the normal controls with less relevant information (34). As well, we observed that some AD patients had negative mood changes due to the frustration and irritation associated with forgetting words when trying to describe the image. AD speech has higher variation in frequencies (jitter) and amplitude in decibels (shimmer) appears, and more speech pauses. With this criterion, the dataset was split into 3 stages of AD with 1 being the early stage and 3 being the late stage. For both AD/CN and Stages AD/CN classifications, three classification models were considered: Logistic Regression, SVM and Random Forest. For the 2-class classification (AD/CN), the AUC-ROC curve of a test can be used as a criterion to measure the test's discriminative ability, providing information on how good the test is in each clinical situation. The closer an AUC-ROC curve is to the upper left corner, the more efficient the test being performed will be. Two former measures with many distinct thresholds for the logistic regression can be generated and plotted on a single graph to merge the False Positive Rate and the True Positive Rate into a single metric. The resulting curve metric we consider is the area under this curve, which is the AUC-ROC graph. Based on the ROC curves of the models, the one which belongs to the Random Forest is the nearest curve to the ideal one, so this model has the better performance. Random forest is an example of ensemble learning in which many models are fitted to distinct subsets of a training dataset and the predictions from all models are then combined. It solves the over-fitting problem and acquires techniques for balancing error in unbalanced data sets of classes studied (38). Based on the confusion matrix of the Random Forest model in 2 class classification (AD/CN), the predictions of the first class (CN) have 15% errors and for the AD class, it is 20% which shows that the model predicts CN samples better than the AD ones. Results show that the model is best performing for the stage 1 class and has the poorest performance for the stage 2 class. It was discovered based on the Random Forest model that jitter and amplitude in decibels (shimmer) are higher in AD speech, and there are more speech pauses. Jitter is the most important feature and helps the model perform much better when predicting the classes. But on the other hand, the number of silence parts has the least impact in classification, the reason being that the audio files were split into multiple parts, so this feature has lost its meaning. If the model were managed without data augmentation and the files were split into smaller parts, this feature would play a much more important role.

The model can be used to screen and successfully distinguish between sick and healthy individuals and can further be applied to distinguish the stage of the disease. This framework can also accommodate any future changes, such as improvement to the generality of the classification results by using larger speech databases with a greater number of speakers. Implementing this diagnostic model to a telemedicine platform would provide patients with limited mobility and/or geographic limits access to specialists, making medical consultations and diagnostics more affordable and convenient (39). Further procedures and tests such as performing a physical exam, laboratory tests, neurological exam, mental cognitive status tests, and brain imaging can be used to confirm the results and to determine the stage of Alzheimer's or the patient's state of mental health. However, patient engagement and early intervention would be aided by a telemedicine platform.

## Data Availability

Publicly available datasets were analyzed in this study. This data can be found here: The dataset can be requested and accessed from Alzheimer's Dementia Recognition through Spontaneous Speech (ADReSS), INTERSPEECH 2020 - Alzheimer's Dementia Recognition through Spontaneous Speech: The ADReSS Challenge. (n.d.). Retrieved March 17, 2022, from http://www.homepages.ed.ac.uk/sluzfil/ADReSS/.
